# The COVID-19 legacy: consequences for the human DNA methylome and therapeutic perspectives

**DOI:** 10.1007/s11357-024-01406-7

**Published:** 2024-11-05

**Authors:** Carlo Gaetano, Sandra Atlante, Michela Gottardi Zamperla, Veronica Barbi, Davide Gentilini, Barbara Illi, Marco Malavolta, Fabio Martelli, Antonella Farsetti

**Affiliations:** 1 Laboratory of Epigenetics, Istituti Clinici Scientifici Maugeri IRCCS, 27100 Pavia, Italy; 2 Institute for Systems Analysis and Computer Science, National Research Council (CNR)-IASI, 00185 Rome, Italy; 3 Department of Brain and Behavioral Sciences, University of Pavia, 27100 Pavia, Italy; 4 Bioinformatics and Statistical Genomics Unit, IRCCS Istituto Auxologico Italiano, 20095 Cusano Milanino, Italy; 5 Institute of Molecular Biology and Pathology, National Research Council (CNR), c/o Sapienza University of Rome, 00185 Rome, Italy; 6 Advanced Technology Center for Aging Research and Geriatric Mouse Clinic, IRCCS INRCA, 60121 Ancona, Italy; 7 Laboratory of Molecular Cardiology, IRCCS Policlinico San Donato, 20097 Milan, Italy

**Keywords:** Coronavirus, Epigenome, Precision medicine, Rehabilitation, Long COVID-19, Aging

## Abstract

The COVID-19 pandemic has left a lasting legacy on human health, extending beyond the acute phase of infection. This article explores the evidence suggesting that SARS-CoV-2 infection can induce persistent epigenetic modifications, particularly in DNA methylation patterns, with potential long-term consequences for individuals’ health and aging trajectories. The review discusses the potential of DNA methylation-based biomarkers, such as epigenetic clocks, to identify individuals at risk for accelerated aging and tailor personalized interventions. Integrating epigenetic clock analysis into clinical management could mark a new era of personalized treatment for COVID-19, possibly helping clinicians to understand patient susceptibility to severe outcomes and establish preventive strategies. Several valuable reviews address the role of epigenetics in infectious diseases, including the Sars-CoV-2 infection. However, this article provides an original overview of the current understanding of the epigenetic dimensions of COVID-19, offering insights into the long-term health implications of the pandemic. While acknowledging the limitations of current data, we emphasize the need for future research to unravel the precise mechanisms underlying COVID-19-induced epigenetic changes and to explore potential approaches to target these modifications.

**Graphical Abstract: Impact of SARS-CoV-2 infection on the epigenetic landscape and individual response:**

Following SARS-CoV-2 infection, individuals may develop either a normal immune response or an aberrant one, such as a cytokine storm. Both scenarios can result in long-lasting consequences, known as “long COVID.” This condition can reshape the epigenetic landscape by altering DNA methylation patterns, contributing to the “epigenetic drift.” This drift, further influenced by various factors, can lead to changes in gene expression, immune functionality, and disease susceptibility. One significant consequence of the epigenetic drift is the acceleration of biological aging, which can profoundly impact personalized medical interventions. Created with BioRender.com.

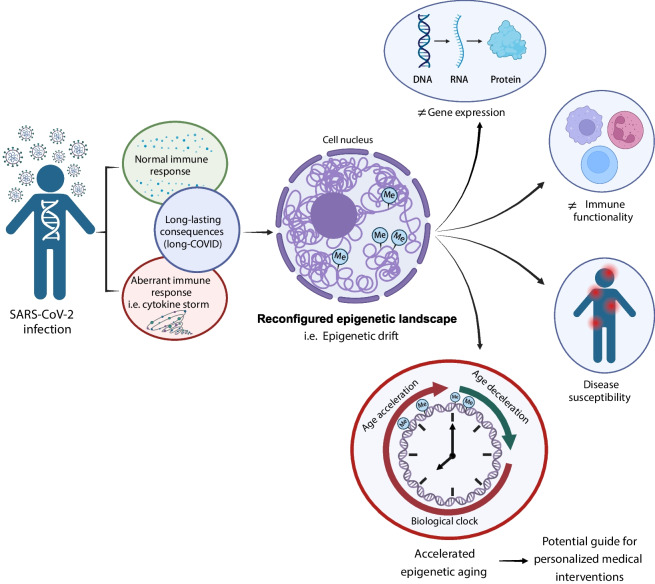

**Supplementary Information:**

The online version contains supplementary material available at 10.1007/s11357-024-01406-7.

## Introduction

As the world gradually emerges from the shadow of the COVID-19 pandemic, a new challenge arises in understanding its lasting impact—not only on global health systems and economies but, as a body of literature suggests, on the human epigenome. The field continues to evolve, with research on these topics actively expanding since the pandemic began. As of September 2024, the PubMed database lists half a million publications related to COVID-19 published since 2020. Among these, approximately one thousand explore the role of epigenetics in the pathophysiology of COVID-19 syndrome, with about 57% of them published since 2022. Comparable results were obtained by exploring Scopus and the Web of Science repositories (see Table S1).

However, comprehensive statistics are still developing as new papers emerge in high-impact journals exploring how epigenetic mechanisms influence the disease, immune responses, and potential therapies, suggesting overall how crucial it is for researchers to follow these developments, as they could provide critical insights for future therapeutic strategies against not just COVID-19 but other viral diseases as well.

The encounter with SARS-CoV-2 has propelled us into a new era of genomic inquiry, with emerging research suggesting that the interaction between the virus and its human host leaves a trail of epigenetic modifications, particularly in DNA methylation [1–3]. These changes open a window to understanding how viral infections can have long-lasting genetic consequences, influencing gene expression, modulating immune responses, and potentially predisposing individuals to various health conditions long after the acute phase of the infection has passed. Researchers have noted alterations in methylation patterns associated with immune function and inflammatory responses, critical factors in the body’s defense against COVID-19 [4, 5].

These epigenetic changes have sparked a discussion in the scientific community about the broader implications for long-term health. Survivors of COVID-19, often struggling with lasting effects known as “long COVID,” may face a reconfigured epigenetic landscape. Studies suggest that the methylation changes might contribute to persistent symptoms and complications, marking a significant move in our understanding of post-viral syndromes [6, 7].

Exploring the consequences of the COVID-19 epigenetic legacy might open new avenues for therapeutic interventions. By dissecting the mechanisms through which SARS-CoV-2 alters human DNA methylation patterns, we can envision targeted treatments that address these molecular changes, potentially mitigating the long-term impacts of the virus. This article aims to synthesize the current knowledge in this rapidly evolving field, highlighting the intricate connections between a global health crisis and the subtle yet profound alterations in the human genome epigenetic framework.

## Selection criteria and methodology

This review article aims to highlight the potential epigenetic consequences of COVID-19, drawing researchers’ and clinicians’ attention to alterations that may not be readily apparent through conventional clinical investigations. While not a systematic review, it offers a focused perspective on the impact of SARS-CoV-2 infection on the human epigenome, particularly DNA methylation.

We curated relevant studies primarily through a PubMed search using keywords tailored to each section of this manuscript (see Supplementary Table S2 for details). We focused on peer-reviewed articles published in English between 2020 and 2024, prioritizing research investigating the effects of SARS-CoV-2 infection on the human epigenome. Cross-referencing and expert recommendations aided in identifying additional pertinent articles.

The final selection of articles was based on their relevance and potential to enhance our understanding of COVID-19 epigenetic implications. We carefully evaluated the information extracted from these studies to ensure an accurate representation of the original research findings. This synthesized information offers an overview of current knowledge in this rapidly evolving field. Due to the volume of research in this area, we could not include every relevant article, and we apologize to the authors whose work could not be incorporated into this review.

## DNA cytosine methylation

DNA cytosine methylation, specifically at the fifth position of the cytosine base (5mC), is a cornerstone of the DNA methylome and a fundamental aspect of the human epigenome. This process involves adding methyl groups primarily to cytosine bases next to guanine, known as CpG sites, and is instrumental in modulating gene expression without altering the DNA sequence. DNA methylation patterns are regulated by DNA methyltransferases (DNMTs), which add methyl groups, and Ten-Eleven Translocation (TET) enzymes, which actively demethylate DNA [8]. These patterns can change in response to environmental factors, aging, and disease states, reflecting an individual’s biological history and health status [9].

The regulation of the DNA methylome is a complex process influenced by a network of factors, including genetic predisposition, environmental exposures, and lifestyle choices. DNMTs and TETs are at the forefront of this regulatory mechanism, orchestrating the addition and removal of methyl groups in response to internal and external stimuli. The balance these enzymes maintain is critical for normal development and cellular function, with aberrations often linked to various pathologies, including cancer, neurological disorders, and autoimmune diseases [8, 10]. Thus, the DNA methylome acts as a crucial mediator between the genome and the environment, translating external signals into genomic responses with lasting impacts on gene expression and cellular behavior [11].

## DNA methylation and viral diseases

In clinical practice, the implications of DNA methylation in disease pathogenesis have become increasingly evident in various human pathophysiological conditions. Aberrant methylation patterns are linked to a spectrum of pathologies, ranging from cancers to autoimmune disorders and neurological conditions. In oncology, hypermethylation of tumor suppressor genes is a well-documented phenomenon contributing to tumorigenesis and cancer progression [12]. Similarly, in autoimmune diseases, hypomethylation of specific genes can lead to dysregulated immune responses [13].

The role of DNA methylation becomes particularly intriguing in the context of viral infections [14]. Viruses can induce epigenetic changes in host cells, affecting the expression of both viral and host genes. For instance, the Epstein–Barr virus (EBV) has been shown to induce methylation alterations in host cells, contributing to malignancies such as Burkitt’s lymphoma [15]. The human papillomavirus (HPV) similarly utilizes methylation changes to evade immune detection and establish persistent infections, which can lead to cervical and other cancers [16, 17]. Coronaviruses such as the SARS-CoV, MERS-CoV, and influenza viruses also induce methylation changes, particularly in immune-related genes primarily driven by the immune response to infection [18, 19]. However, influenza virus changes tend to be more transient than those observed in coronaviruses, and they usually revert to baseline post-recovery. In contrast, some coronavirus alterations can persist long-term, contributing to post-viral syndromes, such as long-term respiratory issues, e.g*.*, asthma [20–22].

Also, non-respiratory viral infections demonstrate distinct patterns of DNA methylation alterations that can have long-term consequences for the host.

The Human Immunodeficiency Virus (HIV) infection is associated with broad changes in the DNA methylome, particularly in immune-related genes. HIV-induced chronic immune activation has been linked to hypermethylation of Interferon-gamma (*IFN-γ*) and other immune signaling genes, contributing to immune dysregulation and persistent inflammation. These methylation changes are thought to exacerbate HIV-associated comorbidities [23, 24].

Cytomegalovirus (CMV), a common herpesvirus, induces significant epigenetic changes in the host. Chronic CMV infection in elderly populations leads to an “immunosenescent” phenotype, partly due to altered DNA methylation patterns in genes related to immune surveillance and function. In individuals with compromised immune systems, CMV-induced methylation changes have been implicated in the progression of various malignancies, including glioblastoma and colorectal cancer [25].

Hepatitis B Virus (HBV) and Hepatitis C Virus (HCV) are known to induce methylation alterations that contribute to the development of liver cancer. In chronic HBV infection, hypermethylation of the p16 and RASSF1A tumor suppressor genes is frequent, which promotes hepatocellular carcinoma (HCC) development. Similarly, HCV has been associated with DNA hypermethylation of genes involved in apoptosis and immune regulation, contributing to hepatocarcinogenesis [26, 27].

These examples highlight how viruses can hijack the host epigenetic machinery for their benefit, ultimately leading to disease. In the case of COVID-19, SARS-CoV-2 has been implicated in altering DNA methylation patterns, though the full extent and long-term consequences of these changes are still under investigation. Initial studies suggest that these methylation alterations could have implications for the severity of the infection and the risk of long-term complications. For example, changes in methylation patterns have been associated with immune response dysregulation in COVID-19 patients, potentially contributing to the cytokine storms observed in severe cases [28, 29]. Genes involved in cytokine signaling and inflammation, such as Interleukin 6 (*IL-6*), Tumor Necrosis Factor-alpha (*TNF-α*), Interferon Alpha/Beta Receptor 2 (*IFNAR2*), and 2’-5’-Oligoadenylate Synthetase 1 (*OAS1*), exhibit differential methylation in severe cases compared to mild or moderate cases [30, 31].

Furthermore, severe COVID-19 patients often exhibit hypomethylation at specific loci, which can lead to hyperactivation of pro-inflammatory genes [31]. Additionally, in severe COVID-19 patients, it has been observed a differential methylation in the promoters of the Angiotensin Converting Enzyme 2 (*ACE2*) receptor gene, which the SARS-CoV-2 virus exploits to enter host cells, and of the Transmembrane Serine Protease 2 (*TMPRSS2*), that processes it [32, 33]. Indeed, an altered methylation in these regions can influence the expression of these entry factors, possibly affecting viral load and disease severity. These markers were involved in pathways related to immune response, inflammation, and viral defense mechanisms, suggesting that methylation profiling could potentially serve as a biomarker for COVID-19 severity [18, 34]. Moreover, severe cases show methylation patterns that resemble those of older individuals, which may contribute to the increased vulnerability of the elderly to severe outcomes from COVID-19. These epigenetic changes reflect a stressed immune system and an elevated inflammatory response [35].

For clinicians, recognizing the role of DNA methylation in disease can enhance the understanding of pathogenesis, inform diagnostic strategies, and guide more targeted treatments. As our knowledge of epigenetics in viral infections expands, it offers the potential to refine our approach to managing these diseases, providing more insights into their impact on human health [14, 36, 37].

## The epigenetic drift

**Fig. 1:**
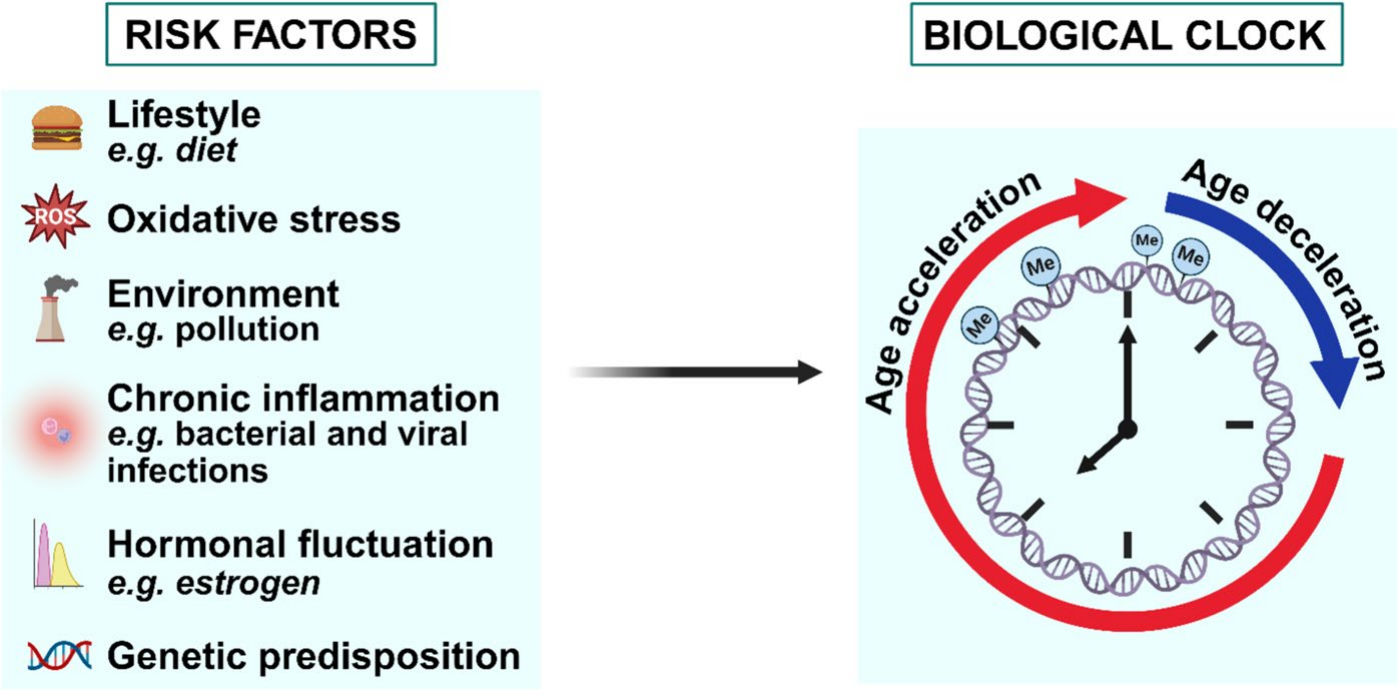
**Multiple factors influence biological aging, contributing to epigenetic drift.** This image illustrates various contributors to epigenetic drift and their effects on biological aging. Epigenetic drift refers to the gradual changes in DNA-epigenetic marks that accumulate over time, influenced by internal and external factors. These factors include genetic predisposition, hormonal fluctuations, oxidative stress, chronic inflammation, lifestyle choices, and exposure to environmental pollutants. Created with BioRender.com

Alterations in 5mC patterns are part of the so-called “epigenetic drift,” which refers to the gradual changes in the epigenetic marks on an individual DNA accumulating during aging [38]. Unlike genetic mutations, which involve changes in the DNA sequence itself, epigenetic changes affect how genes are expressed without altering the underlying DNA sequence. These alterations represent a divergence, accumulating with age, regarding the DNA methylation patterns observed in younger individuals. From a physiopathological perspective, epigenetic drift is considered to contribute to the aging phenotype and potentially affect health and disease susceptibility—a phenomenon defined as gradual, extensive [38–40] and influenced by a variety of factors including i) the intrinsic genetic makeup of an individual where specific genes may predispose individuals to more rapid or pronounced epigenetic changes with age [41]; ii) environmental factors such as pollutants [42]; iii) chronic inflammation, releasing inflammatory cytokines and other mediators leading to DNA methylation and histone modification changes [43]; iv) hormonal fluctuations, especially those associated with aging (like changes in estrogen and testosterone levels) [44]; v) nutrients and dietary components such as folate and vitamin B12 are crucial for the methylation process -indeed deficiencies or excesses in certain nutrients can alter DNA methylation patterns- [45]; vi) increased oxidative stress, which occurs during aging, can damage DNA and affect epigenetic regulation, contributing to drift [46] (Fig. 1).

Notably, epigenetic alterations during aging can be classified into two categories: random epigenetic drift (stochastic modifications) and directed/imposed epigenetic aging changes. The first ones, “stochastic modifications”, are unpredictable DNA methylation changes that accumulate with age and do not follow a specific pattern [47]. These alterations can result from environmental influences, cellular stress, or errors in the maintenance of methylation during cell division due, for instance, to the epigenetic machinery malfunctioning (*e.g.*, DNA methyltransferases), which can lead to sporadic gains or losses of methylation at various genomic sites, contributing to cellular heterogeneity [48]. Random epigenetic drift contributes to age-related dysfunction and is implicated in diseases such as cancer, where stochastic methylation alterations can lead to inappropriate gene activation or silencing [49]. Meanwhile, directed/imposed epigenetic changes are systematic, reproducible, and usually occur in specific genomic regions. During aging, they are often part of a programmed process where specific genes undergo predictable modulations in DNA methylation as part of biological aging, affecting regulatory pathways that control cell cycle, metabolism, development, growth, and senescence. Genetic factors influence this direct regulation and are more consistent across individuals, reflecting a coordinated aging program [50]. They can serve as reliable biomarkers of biological age: Horvath et al*. *[51] demonstrated that specific, age-related methylation changes can accurately predict biological age, indicating that certain epigenetic modifications are not random but directed by underlying biological processes. This distinction is crucial for understanding the aging process and its implications for health and disease. While random epigenetic drift is characterized by non-specific, stochastic changes in methylation across the genome, driven by random errors or environmental factors, directed epigenetic aging changes are consistent, systematic, and occur predictably, reflecting an organized biological aging process [52, 53]. The interplay between these two types of changes shapes the epigenetic landscape during aging and contributes to the complex phenotype associated with the aging process.

Indeed, epigenetic drift is a complex process influenced by multiple factors, and its exact mechanism can vary significantly among individuals. Understanding epigenetic drift is crucial as it has implications for aging, developing age-related diseases, and individual variations occurring during aging, contributing to changes in our internal biological clock [54, 55]. Moreover, developing interventions that may be exploited to slow down or reverse the aging process is essential [56].

## Definition of the epigenetic biological clock and implication in the long COVID-19 syndrome

The concept of an epigenetic clock represents a significant advancement in the field of aging research with potential implications for clinical practice. This clock is an epigenetic biomarker of aging, determined primarily by changes in DNA methylation patterns of several hundred CpG sites at specific sites in the genome, which tend to undergo predictable changes as individuals age. Developed by Steve Horvath at UCLA, this method provides a remarkably accurate measure of biological age, which often differs from chronological age and offers insights into an individual’s health and longevity [51]. The epigenetic clock is not merely a marker of the passage of time; it reflects the cumulative impact of an individual’s lifestyle, environment, and biological processes on his/her epigenome. Studies have shown that accelerated epigenetic aging, as indicated by this clock, is associated with various age-related diseases, including cancer, cardiovascular disease, and neurodegenerative disorders [57].

**Fig. 2:**
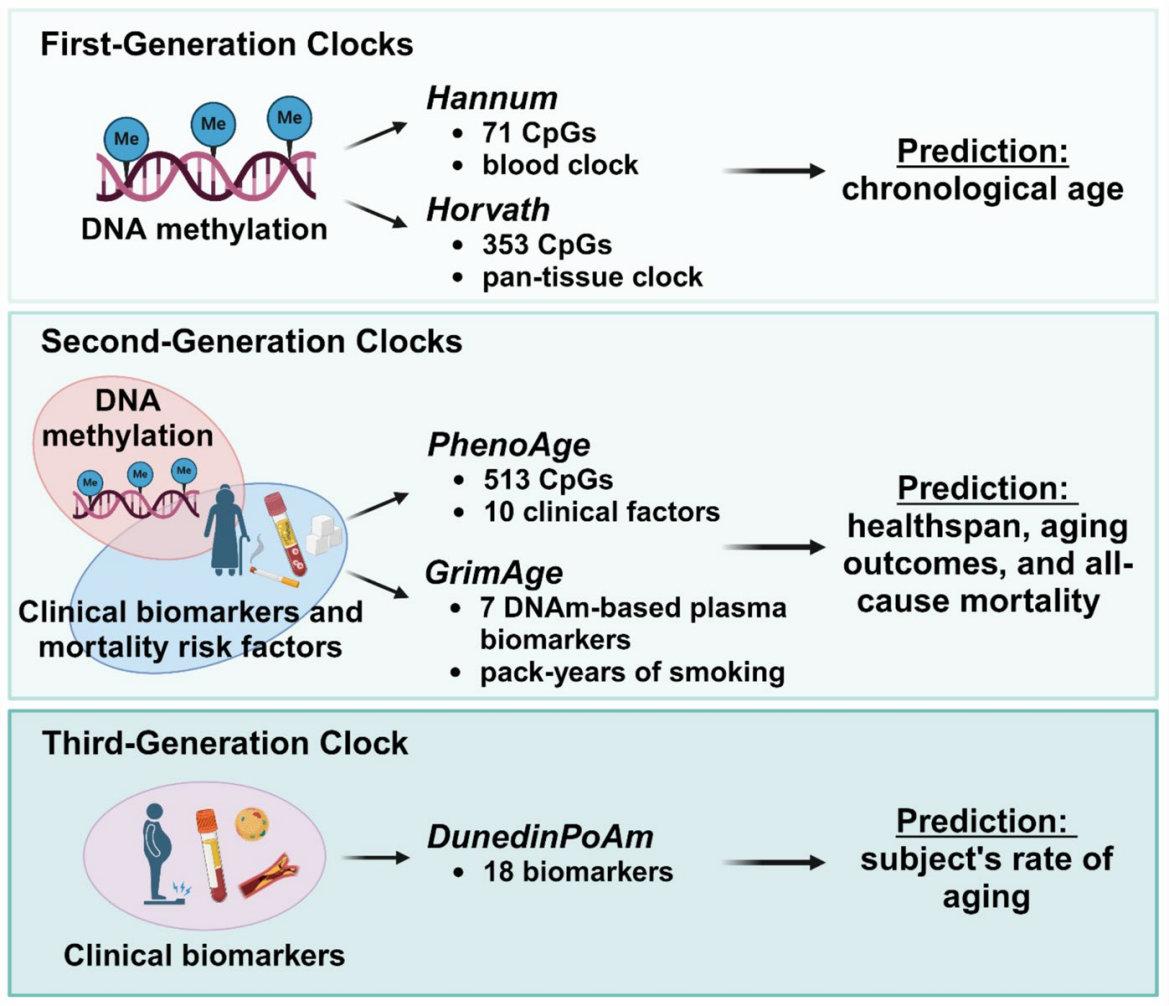
**Evolution of epigenetics clocks. **The image shows the evolution of epigenetic clocks since their inception, progressing through three distinct generations. First-generation clocks, like Horvath and Hannum clocks, predict chronological age using DNA methylation markers. Second-generation clocks, such as PhenoAge and GrimAge, incorporate clinical biomarkers to estimate health span and predict age-related health risks. Finally, third-generation clocks, like DunedinPoAm, focus on tracking the rate of biological aging, offering deeper insights into the relationship between aging, health, and lifestyle. Created with BioRender.com

Recently, the number of algorithms defining specific epigenetic clocks has expanded significantly since its initial development, leading to a growing number of clocks that utilize various aging features [58, 59]. Specifically, epigenetic clocks have evolved through three main generations (Fig. 2), each with distinct features that assess biological age [52, 60]. The so-called first-generation clocks, such as the Horvath and Hannum clocks, primarily rely on DNA methylation markers at specific CpG sites to predict chronological age. Second-generation clocks, like PhenoAge and GrimAge, incorporate additional clinical biomarkers and measures of mortality risk to estimate health span and predict age-related health outcomes such as disease risk, morbidity, and mortality [60]. Third-generation clocks, exemplified by DunedinPoAm, focus on tracking changes in biological age over time, providing insights into the pace of aging and its relationship with health and lifestyle factors [61]. In the context of viral infections, the epigenetic clock has garnered interest for its potential to reveal the impact of diseases like COVID-19 on biological aging processes [3]. Understanding these dynamics is crucial, as it could lead to the development of interventions to mitigate the impact of such infections on epigenetic aging and overall health [62].

Recent studies have shown that even mild COVID-19 infections can impact epigenetic aging. In a Mexican cohort of older adults, GrimAge, a second-generation epigenetic clock associated with mortality and morbidity risk, was significantly elevated in individuals who experienced mild COVID-19 symptoms. This evidence suggests that even mild infections may have long-term consequences for health and aging, potentially mediated by immune system changes and DNA damage [63].

A body of evidence highlighted the negative effect of the SARS-CoV-2 infection on the epigenetic clock in particular. The “long COVID” phenomenon, characterized by persistent symptoms such as fatigue, cognitive impairment, and respiratory difficulties, further complicates understanding COVID-19 epigenetic impact. These prolonged symptoms could be manifestations of lasting epigenetic modifications, indicating a sustained effect of the virus on the patient’s biological age far from the acute phase of the infection [64]. The implications of these findings are substantial, suggesting that the prolonged consequences of COVID-19 syndrome potentially lead to increased biological aging and risk of developing associated age-related diseases.

The intense physiological stress, systemic inflammation, and the body’s response to the viral infection are hypothesized to cause alterations in the DNA methylation patterns in COVID-19-affected individuals, potentially leading to an accelerated aging process. Studies have suggested that the severe inflammatory response, characterized by the release of pro-inflammatory cytokines and oxidative stress associated with COVID-19, can induce epigenetic changes, which may be reflected in the acceleration of the epigenetic clock [54]. Epigenetic clocks like GrimAge have been shown to correlate well with mortality risk and may be used to identify individuals whose biological age has accelerated post-infection [63]. Epigenetic clocks, which measure methylation at specific CpG sites, can potentially detect accelerated aging in COVID-19 patients, characterized by a significant difference between the chronological and the biological age. This approach offers a way to monitor long-term health risks in COVID-19 survivors, particularly those at higher risk for age-related diseases. The potential of the epigenetic clock to act as a predictive biomarker for COVID-19 severity has been a subject of keen interest. The correlation between advanced biological age, as indicated by the epigenetic clock, and increased severity of COVID-19 symptoms underscores the role of biological aging in the body’s response to the virus. This relationship is particularly evident in older populations, who generally experience more severe disease manifestations, likely due to a more “aged” epigenome [62]. This insight opens the door to personalized medicine approaches, where understanding an individual’s epigenetic age could inform clinical decisions and treatment strategies. For instance, in age-related diseases, such as type 2 diabetes or various types of cancer, exploiting the epigenetic regulation of different genes responsible for interindividual variability in the response to antidiabetic drugs or chemotherapies might lead to better outcomes [65, 66].

Furthermore, it is crucial to recognize that epigenetic clocks, while powerful tools for assessing biological age, are not immune to the influence of social determinants of health. Socioeconomic status (SES), encompassing factors like income, education, and occupation, has emerged as a critical modulator of epigenetic aging [67, 68]. Studies have consistently shown that individuals from lower socioeconomic backgrounds often exhibit accelerated epigenetic aging compared to their more affluent counterparts. This disparity can be attributed to the cumulative impact of various factors associated with low SES, including limited access to healthcare, unhealthy dietary habits, exposure to environmental pollutants, and chronic stress [69]. These factors can trigger epigenetic changes, contributing to accelerated biological aging and increased susceptibility to age-related diseases. The intricate relationship between socioeconomic conditions and epigenetic aging underscores the importance of considering social determinants when interpreting epigenetic clock data and developing interventions to promote healthy aging [68, 70, 71].

In light of these observations, clinical epigenetic interventions that target the specific epigenetic alterations caused by COVID-19 or other infectious diseases could be of relevant social interest. Such interventions could address the virus acute symptoms and potentially mitigate its long-term impacts on the aging process. This approach represents a paradigm shift in treating infectious diseases, where the focus extends from immediate symptom relief to long-term health maintenance and aging management [72, 73]. Although not yet fully understood and not mainly considered in the clinical context, the epigenetic clock might offer clinicians a window into the biological impact of their patients’ environmental and lifestyle factors and diseases. It is a tool for assessing biological aging and a potential guide for personalized medical interventions that could slow the epigenetic aging process, improving health outcomes and extending a healthy lifespan [74].

**Fig. 3:**
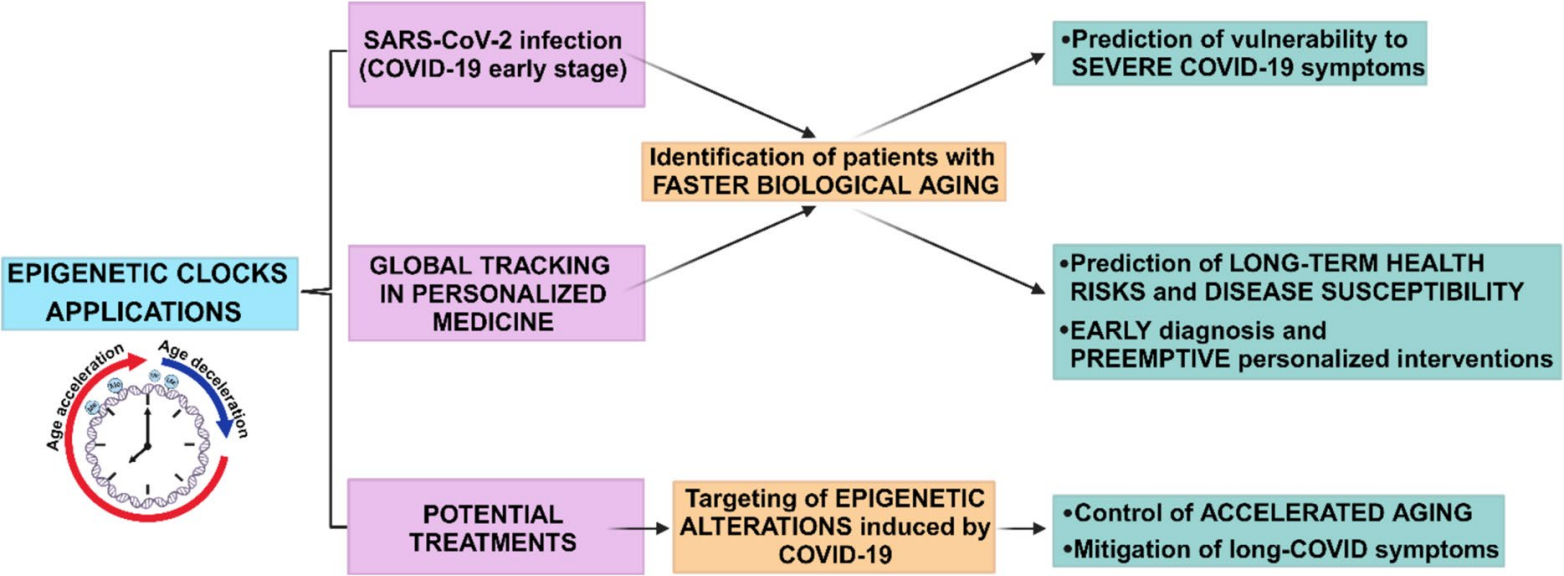
**Epigenetic clocks applications.** The image shows the possible applications of epigenetic clocks in COVID-19 context. During early SARS-CoV-2 infection, these clocks can help assess the biological impact of the disease; identifying patients with accelerated biological aging may help in the prediction of worse outcomes, such as severe symptoms like the cytokine storm. This could be helpful also in predicting long-term health risks and disease susceptibility, providing the tools for early diagnosis and preemptive personalized medicine. Finally, epigenetic alterations caused by COVID-19, including accelerated biological aging, could be potentially treated with epigenetic enzyme inhibitors, such as DNA methyltransferase inhibitors. Created with BioRender.com

In particular, applying epigenetic clocks might provide a promising framework for assessing biological aging and susceptibility to various diseases, including COVID-19 [75]. These clocks could be integrated into the clinical management of COVID-19 or other diseases to monitor disease severity and identify individuals at heightened risk for severe outcomes. However, the timing of the clock application could be crucial and vary based on the clinical queries (Fig. 3).

1. At the time of infection: Generating epigenetic clocks during the early stages of COVID-19 may help clinicians gauge the immediate biological impact of the disease. By assessing the biological age compared to the chronological age, clinicians can identify patients with a faster biological aging trajectory, potentially linked to worse outcomes [76, 77]. For instance, accelerated biological aging due to infection-related inflammation and oxidative stress could exacerbate the patient’s vulnerability to severe symptoms, such as the cytokine storm often observed in severe COVID-19 cases [29, 35].
2. Global tracking in personalized medicine: Beyond infection, tracking individuals’ epigenetic clocks in the broader context of personalized medicine offers valuable insight into long-term health risks and disease susceptibility [19, 78]. Monitoring the epigenetic clock over time could reveal individuals whose biological aging progresses faster due to lifestyle, environmental exposures, or infections like COVID-19 [79, 80]. This approach can aid in the early identification of health vulnerabilities, enabling preemptive interventions, such as personalized therapies targeting inflammation, stress reduction, and pharmacological agents like DNA methyltransferase inhibitors to slow down epigenetic aging [19, 22].
3. Potential treatments: Various therapeutic strategies could target the epigenetic alterations caused by COVID-19. DNA methyltransferase inhibitors such as Azacitidine and Decitabine, used in oncology, could be repurposed to address the methylation changes observed in COVID-19 patients [22]. In the future, activatory intervention could also be envisaged [81]. Theoretically, these treatments might control the accelerated aging process observed in severe cases and mitigate the long-term consequences of COVID-19, including “long COVID” symptoms. Additionally, histone deacetylase inhibitors, another class of epigenetic modulators, can potentially reduce inflammation and other age-related changes [19, 56, 79] (see the section below about “Reverting the clock”).

Table 1 summarizes the related literature in the manuscript addressing the issue of biological clock, DNA methylation, and COVID-19.

**Table 1:** Epigenetic impact of COVID-19

COVID-19 population	Findings	Epigenetic clock	Methods	Ref
9 severe COVID-19, 5 influenza, and 9 co-infected patients vs* 9* healthy controls	DNA methylation signature associated with severe COVID-19 and mortality risk	GrimAge	Blood samples EPIC array	[82]
21 healthy vs 14 mild or severe COVID-19 patients	Differentially methylated genes related to biological processes, signal transduction, and the immune system	N/A	Blood samples EPIC array	[1]
194 mild vs 213 severe COVID-19 patients	DNA methylation signature (EPICOVID) associated with severity	Hannum Horvath PhenoAge Gonseth-Nusslé	Blood samples EPIC array CpGs pyrosequencing	[34, 83]
63 healthy vs 50 mild and 50 severe COVID-19 patients	Hypomethylation in inflammatory genes, promoter hypermethylation profile correlating with cytokine storm severity	N/A	Blood samples EPIC array	[31]
144 COVID-19-free vs 117 post-COVID-19 patients	Significant DeltaAge acceleration	Bekaert	CpGs pyrosequencing	[3]
U.K. Biobank England (613 individuals), 154 deceased COVID-19 patients	Aging biomarkers associated with long-term vulnerability to COVID-19	PhenoAge	Biological samples	[76]
47 severe COVID-19 patients, 27 with ARDS	No evidence of accelerated bAge in severe COVID-19 patients	3 age-associated regions sequencing Horvath Hannum Skin-Horvath	Blood samples	[84]
296 healthy vs 164 mild and severe COVID-19 patients	Differentially methylated CpGs associated with SARS-CoV-2 infection; development of a classification model for prediction of disease severity	N/A	Blood samples EPIC array	[18, 85]
232 controls vs 48 mild or severe COVID-61 patients	Dynamic acceleration of epigenetic aging across COVID-19 disease phases	Horvath Hannum Skin-Horvath PhenoAge GrimAge	Blood samples EPIC array	[2]
21 patients Pre-COVID-19 vs post-COVID-19 infection	PhenoAge and GrimAge significantly increase in people over 50 following COVID-19 infection	Horvath Hannum Skin-Horvath PhenoAge GrimAge	Blood samples EPIC array	[86]
38 healthy vs 101 mild and severe COVID-19 patients	Methylation patterns differ in severe cases, especially in immune response-related signaling pathways	N/A	Biological samples	[87]
73 healthy vs 473 mild and severe COVID-19 patients	Differentially methylated regions, hypomethylation of IFI44L	Hannum	Blood samples EPIC array	[88]
COVID-19 outcomes from different studies	No clear association between aging and susceptibility to COVID-19; COVID-19 severity has a negative causal relationship with the GrimAge clock	PhenoAge GrimAge Intrinsic Horvath Hannum	Mendelian randomization	[89]
101 healthy vs 473 mild and severe COVID-19 patients	Differentially methylated CpG sites shared between severe and mild cases, mainly associated with interferon signaling pathway and B/T lymphocytes hyper-activation	N/A	Blood samples EPIC array	[28]
33 non-COVID-19 vs 100 severe COVID-19 patients (ARDS)	Epigenetic signature in severe COVID-19 patients that predicts the clinical outcome in immune-response pathways	N/A	Blood samples EPIC array	[30]
123 mild vs 64 severe COVID-19 patients	Epigenetic drift and age acceleration associated with severe prognosis; identification of a specific signature able to discriminate the disease evolution	GrimAge	Blood samples EPIC array	[77]
8 infants exposed to COVID-19 during pregnancy vs 8 control infants with no COVID-19 exposure	COVID-19 induces differential DNA methylation in umbilical cord blood cells	N/A	Umbilical cord blood samples EPIC array	[7]
101 healthy vs 360 mild and 113 severe COVID-19 patients	DNA methylation varies according to COVID-19 severity, influencing immune-response pathways associated with viral infections, and the expression of genes associated with COVID-19 progression	N/A, *feature-ranking algorithms*: LASSO, LightGBM, MCFS *Classification algorithm:* IFS, DT, kNN, RF, SVM	Blood samples Machine-learning workflow	[4]
68 severe vs 19 deceased COVID-19 patients, and 21 controls	Significant accelerated epigenetic aging in severe COVID-19 cases	Horvath PhenoAge GrimAge SkinandBlood	Blood samples EPIC array	[90]
47 mild vs severe COVID-19 patients	Epigenetic age in COVID-19 patients significantly differ from the chronological age, but only GrimAge is elevated in older adults with mild COVID‑19	Horvath Hannum Skin-Horvath PhenoAge GrimAge	Blood samples EPIC array	[63]
27 healthy vs 15 post-COVID-19 and 103 severe (PASC) COVID-19 patients	Differentially methylated genes related to COVID syndrome severity; PASC induces specific DNA methylation of transcription factor motifs	N/A, differential analysis and 2 machine learning algorithms	Blood samples NEBNext enzymatic methyl-seq	[91]
191 control vs 96 long-COVID-19 patients	Accelerated biological aging, and enhanced epigenetic drift detected in long-COVID‑19 patients	Horvath	Blood samples EPIC array	[80]

The table summarizes the relevant studies exploring the relationship between DNA methylation and COVID-19, highlighting populations, main findings, epigenetic clocks, samples, and methods used for DNA methylation profiling, according to publication date. Several epigenetic clocks, which estimate biological age based on DNA methylation patterns, have been used to investigate the potential role of DNA methylation in COVID-19 severity and prognosis

*N/A* not applicable, *ARDS* acute respiratory distress syndrome, *PASC* post-acute sequelae of SARS-CoV-2, *LASSO* most minor absolute shrinkage and selection operator, *LightGBM* light gradient-boosting machine, *MCFS* Monte Carlo feature selection, *IFS* incremental feature selection, *DT* decision tree, *kNN* K-nearest neighbor, *RF* random forest, *SVM* support vector machine

## COVID-19 and epigenetic drift

The COVID-19 pandemic has prompted extensive research into the mechanisms underlying the virus impact on human health. One significant area of focus has been the role of DNA methylation. Recent studies have shed light on the epigenetic dimension of SARS-CoV-2 impact, suggesting that the virus may interact with and influence this epigenetic mechanism. For instance, Calzari et al*. *[77] conducted an epigenome-wide association study, identifying specific markers of severe outcomes in COVID-19 patients. Their findings suggest that changes in DNA methylation could indicate the accumulation of DNA damage related to the progression of COVID-19 infection, potentially serving as a biomarker for predicting disease severity [77].

In this direction, exploring further the epigenome landscape of COVID-19 and examining DNA methylation patterns in the context of the disease offered insights into the origins of the cell-free DNA (cfDNA) detected in COVID-19 patients, which could be critical for understanding the disease progression and impact, highlighting the potential role of epigenetic changes in the tissue response to SARS-CoV-2 infection [92]. The epigenetic aspects of the interaction between SARS-CoV-2 and the human host, including DNA methylation within the virus coding region, have also been explored. Several potentially significant consequences emerged, indicating that changes in DNA methylation patterns in the host due to SARS-CoV-2 infection can alter the viral replication, the host immune response, and the disease progression, influencing the severity of the disease and the clinical outcomes of COVID-19 patients [28].

Of note, the COVID-19 impact on the epigenetic drift might involve random (stochastic) and directed (programmed) changes. Random epigenetic modifications may result from the inflammatory response and cellular stress induced by the SARS-CoV-2 infection, leading to unpredictable methylation pattern alterations [48]. Meanwhile, directed epigenetic modulations could be connected to the immune system-regulated response and aging-related pathways affected by COVID-19, such as those involving inflammatory cytokines. COVID-19 can accelerate biological aging, as observed in severe cases with methylation changes in immune-related genes, usually associated with the aging process, involved in immune senescence, and increased inflammation (inflammaging) [18, 35, 88]. These directed changes are more consistent and suggest a pattern of accelerated aging induced by the virus, while stochastic alterations contribute to variability in individual responses. Indeed, both types of changes might play a crucial role, but directed changes may have a more profound impact on the aging process due to their consistency and relation to critical biological pathways [77].

Overall, these studies highlight the significance of DNA methylation drift in understanding the progression and severity of COVID-19, particularly concerning aging and immune response. The integration of epigenetic research into the study of COVID-19 offers a novel perspective on the virus impact on human health, suggesting a complex interplay between viral infection, human aging, and epigenetic alterations. As our understanding of these relationships deepens, it could lead to more effective strategies for managing and treating COVID-19, particularly in vulnerable populations such as the elderly [93].

## The clinical epigenetics: vision and limits

Clinical epigenetics is a relatively recent branch of medicine that focuses on understanding how epigenetic modifications can influence gene expression and contribute to disease pathology. This field aims to bridge the gap between an individual’s genetic blueprint and environmental factors, lifestyle choices, and disease states that can modify this blueprint reversibly and dynamically. Clinical epigenetics seeks to exploit these epigenetic mechanisms for therapeutic purposes, offering the potential for novel treatment strategies that could reverse aberrant epigenetic modifications associated with various diseases.

Overall, the potential therapeutic impact of clinical epigenetics is vast, offering promising avenues for the prevention, diagnosis, and treatment of a wide range of diseases, including cancer, neurological disorders, cardiovascular diseases, and autoimmune conditions. By targeting specific epigenetic changes that contribute to disease progression, therapies can be developed to modulate gene expression in a precise and controlled manner. This approach not only provides opportunities for personalized medicine tailored to the unique epigenetic landscape of an individual but also offers a means to address diseases that have been resistant to conventional therapies. Furthermore, since epigenetic modifications are reversible, clinical epigenetics promises to restore normal gene function and cellular homeostasis, potentially leading to complete disease remission or significantly improved patient outcomes [94].

The therapeutic implications of clinical epigenetics are profound. Epigenetic therapies can potentially revolutionize the treatment of certain types of cancer [95] and hold promise for other diseases, including rare genetic diseases [96]. DNA methyltransferase inhibitors, such as Azacitidine and Decitabine, are already used in treating myelodysplastic syndromes and certain leukemia [97]. Histone deacetylase inhibitors, like Vorinostat and Romidepsin, have shown efficacy in treating cutaneous T-cell lymphoma and are being explored for other cancers and beyond [98]. The potential of miRNA-based therapies is also being explored, with several candidates in clinical trials for various conditions [99].

Hence, clinical epigenetics appears to be a rapidly evolving field key to understanding the complex interplay between genetics, environment, and disease pathophysiology. However, its advancement is limited by our incomplete understanding of the underlying mechanisms of epigenetic changes. This lack of knowledge hinders our ability to interpret epigenetic modifications and their impact on diseases. Epigenetic changes are complex and context-dependent, making it challenging to discern their specific roles in disease progression and treatment outcomes. Furthermore, the reversibility of epigenetic modifications and their dynamic nature in response to environmental factors and aging add to the complexity. As a result, the translation of epigenetic research into clinical practice is impeded, limiting its application in personalized medicine, disease prognosis, and the development of targeted epigenetic therapies. More comprehensive and integrated research is needed to unravel the complex network of epigenetic mechanisms and their interactions with genetic and environmental factors [100].

Despite these limits, a clinical epigenetic approach might offer novel insights into disease mechanisms, opening avenues for diagnosis, prognosis, treatment, and more personalized and effective medical interventions. This paradigm shift underscores the importance of epigenetic factors in health and disease, highlighting the potential for groundbreaking advancements in medical science and patient care [101, 102]. Other comprehensive reviews have previously addressed this topic, offering valuable insights into the broader landscape of epigenetic modifications and infectious diseases, including COVID-19. Readers seeking a broader perspective on the role of epigenetics in various infectious diseases are encouraged to consult these resources [14, 15, 19–21]

## Reverting the clock

In this section, we focus on the possibility of introducing therapeutic approaches, mainly pharmacological, aimed at modifying the methylome with potential consequences on the human biological age and perspective life span. Groundbreaking evidence was presented in a pioneering study led by Greg Fahy and Robert Brooke of Intervene Immune, Inc. and Steve Horvath at UCLA, suggesting the potential for reversing biological aging in humans. Initiated in 2015, their original TRIIM trial [103] witnessed participants effectively reducing their biological age by more than two years following a year-long treatment regimen. This reduction in biological age was consistently observed across Horvath’s epigenetic clocks and, notably, the GrimAge algorithm. GrimAge stands out for its analysis of DNA methylation changes, offering the most precise evaluation of biological age in humans [104].

Although some results about in vivo rejuvenation suggest that it might be achievable, at least under particular experimental conditions [105], it remains unclear whether DNA methylation and the function of the enzymes controlling it have a causal role in this process. Conclusive evidence is missing on whether a temporary reduction in an individual’s epigenetic clock score directly reduces their risk of experiencing age-related health issues.

However, the possibility of intervening in the DNA methylome modulating the biological clock level remains of great interest.

With this new understanding, integrating the DNA methylome-based epigenetic clock analysis into the clinical management of COVID-19 might allow for an approach to patient care based on individual biological aging profiles. As a biomarker, epigenetic age offers clinicians a better understanding of a patient’s susceptibility to severe outcomes from COVID-19. This understanding is crucial, especially given that accelerated biological aging, as indicated by altered DNA methylation patterns, has been associated with increased vulnerability to the virus and its more severe forms [3, 54]. By identifying individuals with advanced epigenetic aging, healthcare providers might tailor preventive strategies and interventions, such as personalized dietary plans, stress reduction techniques, and targeted pharmacotherapy, to potentially reduce the susceptibility to and severity of infection.

The application of epigenetic modifiers, which have shown promise in oncological and other conditions, opens potential pathways for mitigating the effects of COVID-19 on the epigenetic clock. Drugs like DNA methyltransferase inhibitors could be investigated as repurposed drugs to address the epigenetic alterations induced by the virus, potentially slowing down the accelerated aging process and improving overall health outcomes [97]. Furthermore, for patients facing long-term COVID-19 symptoms, more effective rehabilitation strategies could be designed based on their epigenetic age. Such strategies might include targeted physical therapy, cognitive rehabilitation, and mental health support, addressing the comprehensive needs arising from the long-term impacts of COVID-19 on their biological systems [64].

Whether to focus therapeutic interventions on resetting the overall epigenetic clock or targeting specific COVID-19-induced methylation changes remains a complex and unresolved issue [56, 103]. While “rewinding” the biological clock is tempting, the potential risks associated with global hypomethylating agents, such as Decitabine and Azacytidine, warrant caution [106]. Indiscriminate demethylation could inadvertently exacerbate age-related processes, potentially worsening the long-term health consequences of COVID-19.

The current literature suggests that COVID-19 impact on the epigenome involves stochastic changes and potentially directed alterations to specific methylation sites [80]. Therefore, a prudent approach to therapeutic intervention may be necessary.

Instead of broadly reverting the epigenetic clock, future research should prioritize identifying the precise epigenetic marks altered by SARS-CoV-2 infection and developing therapies that can selectively modulate these marks without causing widespread disruptions to the epigenome [18, 30, 33]. This targeted approach could mitigate the long-term health consequences of COVID-19 or other acute or chronic diseases while minimizing the risk of unintended side effects [86].

Moreover, regular monitoring of the epigenetic clock in recovered COVID-19 patients should become a standard part of follow-up care. This approach would enable early detection of accelerated aging or related complications, allowing for timely interventions ranging from lifestyle adjustments to medical therapies. It also underscores the importance of longitudinal studies to understand the full spectrum of COVID-19 impact on biological aging [62]. In addition, the ongoing research into developing new therapeutic agents that can specifically counteract the epigenetic effects of COVID-19 could lead to novel treatments. These treatments would not only target the immediate symptoms of the virus but also counteract its long-term impacts on the aging process, thereby improving the quality of life and longevity of patients worldwide [72].

By embracing a model of care that incorporates the insights provided by the evaluation of the epigenome, clinicians can move beyond a one-size-fits-all approach, offering personalized medical interventions that align with each patient unique biological response to COVID-19. This shift toward personalized epigenetic care might significantly advance our ability to manage the immediate impacts of infectious diseases and their long-term consequences on human health and aging, thereby enhancing the efficacy and precision of clinical interventions in the post-pandemic era [79].

## Conclusions and perspectives

In light of the above considerations, we believe that future research should focus more on: (i) identifying specific COVID-19-induced epigenetic marks, this involves comprehensive epigenomic profiling of COVID-19 patients, comparing their epigenetic landscapes to those of healthy individuals or pre-infection samples - advanced technologies like single-cell epigenomics could refine this analysis by revealing cell-type-specific epigenetic alterations; (ii) understanding the mechanisms of epigenetic dysregulation, investigating how SARS-CoV-2 interacts with and alters the host’s epigenetic machinery is crucial - this knowledge could lead to identifying novel therapeutic targets and developing drugs modulating these pathways; and (iii) developing targeted epigenetic therapies, using the identified epigenetic targets and the underlying mechanisms, novel therapeutic approaches can be developed - this strategy could involve designing drugs targeting the epigenetic enzymes or pathways dysregulated by SARS-CoV-2 infection, utilizing gene editing technologies to precisely modify epigenetic marks, or exploring RNA-based therapies to modulate gene expression.

The development of targeted epigenetic therapies for COVID-19 is a promising but challenging area of research. The current literature, such as the work by Pang et al. [86], highlights the need to move beyond so-called “blunt hammer” approaches and embrace precision epigenetic medicine. By identifying specific COVID-19-induced epigenetic changes and the mechanisms underlying them, we can develop novel therapeutic approaches to mitigate the pandemic long-term health consequences.

While resetting the epigenetic clock might seem attractive, the potential risks and lack of specificity associated with current methods warrant caution. Instead, a more targeted approach focusing on specific COVID-19-induced epigenetic marks to be identified and carefully evaluated, potentially including histone modifications, may offer a more promising avenue for therapeutic intervention. Further research is needed to fully understand the complex epigenetic landscape altered by COVID-19 and develop safe and effective therapies that can address its long-term health implications.

The COVID-19 pandemic has had a long-lasting impact on the human epigenome, mainly through alterations in DNA methylation. This article outlines how the interaction with SARS-CoV-2 might go beyond the immediate immune response, imprinting permanent epigenetic modifications that may influence gene expression, immune functionality, and disease susceptibility in the post-infection period. Exploring the DNA methylome in this context highlights its dynamic regulation by genetic and environmental factors and underscores its pivotal role in disease mechanisms [6, 97].

Longitudinal studies tracking methylation changes over extended periods, even years post-recovery, are crucial to assessing these alterations persistence and impact on long-term health [76, 80, 86].

Furthermore, relying on blood samples for methylation analysis in most research may not fully capture the epigenetic changes in specific tissues or cell types directly impacted by the virus. Cell-type-specific analyses are essential for a more nuanced understanding of COVID-19 epigenetic effects on different organ systems [82]. Additionally, having pre- and post-infection data from the same individuals would offer valuable insights into the trajectory of methylation changes and their correlation with disease severity and long-term complications [86].

Moreover, the current literature may be biased toward individuals with severe COVID-19 symptoms, as they are more likely to be hospitalized and participate in research studies. This aspect could lead to overestimating the epigenetic changes associated with the disease and their impact on aging [107, 108]. Future research should actively recruit and include individuals with a wide range of COVID-19 severities, including those with mild or asymptomatic infections, to provide a more balanced and comprehensive understanding of the epigenetic consequences of the virus.

Future research should also evaluate whether observed DNA methylation patterns are directly attributable to COVID-19 infection or if pre-existing methylation patterns predispose individuals to specific outcomes. Analyzing pre-infection methylation datasets and comparing them to post-infection profiles could help clarify the cause-and-effect relationship between the virus and epigenetic changes.

The long-term relevance of these changes also needs further exploration. Follow-up studies are necessary to determine which cell types retain the epigenetic memory of the infection and if specific cell types, particularly those involved in the adaptive or innate immune response, show distinct methylation profiles in response to viral exposure.

The impact of changes in the DNA methylome on the biological clock, particularly concerning COVID-19, is significant. Evidence suggests that COVID-19 might accelerate epigenetic aging, with the epigenetic clock as a crucial biomarker [2, 54]. This clinical significance implies that the epigenetic clock could assess long-term health risks post-recovery and guide therapeutic interventions [62].

Interestingly, whether multiple COVID-19 infections in a young, healthy individual could lead to accelerated epigenetic aging and a significantly “older” biological clock is intriguing, but a definitive answer is currently lacking [108]. While some studies suggest that severe or mild COVID-19 can accelerate epigenetic aging, the impact of repeated infections on the long-term epigenetic landscape remains unclear. Comprehensive longitudinal studies tracking methylation changes in individuals with multiple infections, particularly in younger populations, are needed to address this question [86].

The heterogeneity of COVID-19-associated diseases underscores the need for more epigenetic-targeted interventions and related technologies, requiring broader cooperation and a multidisciplinary approach. This area has great potential: in particular, the application of epigenetic modifiers in mitigating COVID-19 impact on the epigenome might open an unexplored frontier in managing post-COVID conditions. These therapeutic strategies might range from lifestyle modifications to pharmacological interventions aimed at restoring or maintaining the integrity of the epigenetic landscape [109]. Regular monitoring of the epigenetic clock in recovering COVID-19 individuals might become integral to post-recovery care, aiding in the early detection and management of accelerated aging or related complications. This approach promises to enhance the management of post-COVID health complications and offers a template for addressing future public health crises with an epigenetic dimension. In this light, the interplay between viral infections and the human epigenome will likely remain a critical area of research, driving innovations in treatment, prevention, and healthcare policy [72]. The need for an integrated approach to health that encompasses genetic, epigenetic, and environmental factors shaping human disease and recovery cannot be further postponed.

## Supplementary Information

Below is the link to the electronic supplementary material.

Supplementary file1 (DOCX 393 KB)
