# Correction to: The COVID‑19 legacy: consequences for the human DNA methylome and therapeutic perspectives

**DOI:** 10.1007/s11357-025-01751-1

**Published:** 2025-06-17

**Authors:** Carlo Gaetano, Sandra Atlante, Michela Gottardi Zamperla, Veronica Barbi, Davide Gentilini, Barbara Illi, Marco Malavolta, Fabio Martelli, Antonella Farsetti

**Affiliations:** 1 Laboratory of Epigenetics, Istituti Clinici Scientifici Maugeri IRCCS, 27100 Pavia, Italy; 2 Institute for Systems Analysis and Computer Science, National Research Council (CNR)-IASI, 00185 Rome, Italy; 3 Department of Brain and Behavioral Sciences, University of Pavia, 27100 Pavia, Italy; 4 Bioinformatics and Statistical Genomics Unit, IRCCS Istituto Auxologico Italiano, 20095 Cusano Milanino, Italy; 5 Institute of Molecular Biology and Pathology, National Research Council (CNR), c/o Sapienza University of Rome, 00185 Rome, Italy; 6 Advanced Technology Center for Aging Research and Geriatric Mouse Clinic, IRCCS INRCA, 60121 8 Ancona, Italy; 7 Department of Clinical and Molecular Sciences (DISCLIMO), Università Politecnica delle Marche, 60126 Ancona, Italy; 8 Laboratory of Molecular Cardiology, IRCCS Policlinico, San Donato, 20097 Milan, Italy

**Correction to: GeroScience (2025) 47:483–501**

10.1007/s11357-024-01406-7

In this article the affiliation details for author Marco Malavolta were incorrectly given as “Advanced Technology Center for Aging Research and Geriatric Mouse Clinic, IRCCS INRCA , 60121 Ancona, Italy” but should have been “Advanced Technology Center for Aging Research and Geriatric Mouse Clinic, IRCCS INRCA, 60121 8 Ancona, Italy” and “Department of Clinical and Molecular Sciences (DISCLIMO), Università Politecnica delle Marche, 60126 Ancona, Italy”.

The correct affiliations are now shown in the affiliation section.

