# Supplementary material for: The COVID-19 legacy: consequences for the human DNA methylome and therapeutic perspectives
Source: GeroScience. 2024 Nov 5;47(1):483–501. doi: 10.1007/s11357-024-01406-7 (PMC11872859; doi:10.1007/s11357-024-01406-7)
Supplement: Supplementary file 1 — Supplementary file1 (DOCX 393 KB) [file 11357_2024_1406_MOESM1_ESM.docx]

**Table S1 COVID-19 and Epigenetics publications summary.**

| KEYWORDS | DATABASES | | | |
| --- | --- | --- | --- | --- |
|  | **PERIOD** | **PubMed** | **SCOPUS (all)** | **WoS (all)** |
| COVID-19 | 2020 – 2024 | 444,140 | 1,330,348 | 689,745 |
| COVID-19 and Epigenetics | 2020 – 2024 | 948 | 8,707 | 339 |
| COVID-19 and Epigenetics | 2022 – 2024 | 561 (57%) | 6,786 (78%) | 179 (53%) |

**Table 1 COVID-19 and Epigenetics publications summary.** The table indicates the number of publications related to COVID-19 released since 2020, and the publications released in the period from 2022 to 2024, showing their increase (expressed in percentage) in the last two years. PubMed, SCOPUS and WoS databases were used as sources.

**Table S2 Article research strategy.**

| **REVIEW SECTION** | **ARTICLE** | **PMID** | **KEYWORDS** |
| --- | --- | --- | --- |
| **Introduction** | (Zhou *et al.*, 2021) | **34185889** | (covid-19 OR sars-cov-2) AND (epigenetics) AND (DNA methylation) |
|  | (Cao *et al.*, 2022) | **35440567** | (covid-19 OR sars-cov-2) AND (aging OR ageing) AND (telomere) |
|  | (Mongelli *et al.*, 2021). | **34200325** | (covid-19 OR sars-cov-2) AND (aging OR ageing) AND (telomere) |
|  | (Yuan *et al.*, 2023) | **38062828** | (covid-19 OR sars-cov-2) AND (epigenetics) AND (DNA methylation) |
|  | (Greco *et al.*, 2020) | **33129318** | (covid-19 OR sars-cov-2) AND (cardiovascular) AND (noncoding RNA) |
|  | (Ali *et al.*, 2023) | **37707287** | (covid-19 OR sars-cov-2) AND (vaccination) AND (immunogenicity) and (graft) and (surveillance) AND (transplant) |
|  | (Urday *et al.*, 2023) | **37425024** | (covid-19 OR sars-cov-2) AND (epigenetics) AND (DNA methylation) |
| **DNA cytosine methylation** | (Smith and Meissner, 2013) | **23400093** | (epigenetics) AND ("DNA methylation") AND ("mammalian development") |
|  | (Yuan *et al.*, 2015) | **25692570** | (epigenetics) AND ("DNA methylation") AND ("aging" OR "ageing") |
|  | (Takeshima *et al.*, 2020) | **32663196** | (epigenetics) AND ("DNA methylation") AND (DNMT) AND (TET) |
|  | (Meng *et al.*, 2015) | **25892967** | (epigenetics) AND ("DNA methylation") AND (genome stability) |
| **DNA methylation and viral diseases** | (Esteller, 2008) | **18337604** | ("epigenetics") AND ("cancer") |
|  | (Richardson, 2003) | **14585278** | ("DNA methylation") AND ("autoimmune diseases") |
|  | (Atlante *et al.*, 2020) | **33087172** | (covid-19 OR sars-cov-2) AND (epigenetics) AND (DNA methylation) |
|  | (Niller *et al.*, 2016) | **26659263** | (epigenetics) AND (infection) AND (virus) AND (cancer) |
|  | (Kostareli *et al.*, 2013) | **23635773** | (DNA methylation) AND (infection) AND (virus) AND (cancer OR carcinoma) |
|  | (Zeng *et al.*, 2023). | **37212325** | (DNA methylation) AND (infection) AND (virus) AND (cancer OR carcinoma) |
| **DNA methylation and viral diseases** | (Konigsberg *et al.*, 2021) | **36750622** | (covid-19 OR sars-cov-2) AND (epigenetics) AND (DNA methylation) |
|  | (Crimi *et al.*, 2020) | **32828489** | (DNA methylation) AND (infection) AND (virus) |
|  | (Menachery *et al.*, 2018) | **29339515** | (DNA methylation) AND (infection) AND (virus) |
|  | (Schäfer and Baric, 2017) | **28212305** | (DNA methylation) AND (infection) AND (virus) |
|  | (Patra and Szyf, 2022) | **36002132** | (covid-19 OR sars-cov-2) AND (epigenetics) AND (therapy) |
|  | (Zeng *et al.*, 2020) | **33013899** | (DNA methylation) AND (infection) AND (virus) |
|  | (Friedman *et al.*, 2023) | **36911799** | (epigenetics) AND (immune) AND (plasticity) |
|  | (Yu *et al.*, 2023) | **37424781** | (infection) AND (virus) AND ("carcinogenesis") AND (therapy) |
|  | (Mostafa *et al.*, 2020) | **31630500** | (DNA methylation) AND (infection) AND (virus) AND (cancer OR carcinoma) |
|  | (Telli *et al.*, 2022) | **36177104** | (DNA methylation) AND (infection) AND (virus) AND (cancer OR carcinoma) |
|  | (Barturen *et al.*, 2022) | **35933486** | (covid-19 OR sars-cov-2) AND (epigenetics) AND (infection) AND (cytokine storm) |
|  | (Dey *et al.*, 2023) | **36906872** | (covid-19 OR sars-cov-2) AND (epigenetics) AND (infection) AND (cytokine storm) |
|  | (Bradic *et al.*, 2022) | **36371196** | (covid-19 OR sars-cov-2) AND (epigenetics) AND (DNA methylation) |
|  | (Balnis *et al.*, 2021) | **34034806** | (covid-19 OR sars-cov-2) AND (epigenetics) AND (DNA methylation) |
|  | (Ragia and Manolopoulos, 2020) | **33243086** | (covid-19 OR sars-cov-2) AND (epigenetic) AND (ace2) |
|  | (Daniel *et al.*, 2022) | **36038007** | (covid-19 OR sars-cov-2) AND (epigenetics) AND (DNA methylation) |
|  | (Castro de Moura *et al.*, 2021) | **33867313** | (covid-19 OR sars-cov-2) AND (epigenetics) AND (DNA methylation) |
|  | (Bektas *et al.*, 2020) | **32849908** | (covid-19 OR sars-cov-2) AND (inflammaging) |
|  | (Kgatle *et al.*, 2021) | **34691068** | (covid-19 OR sars-cov-2) AND (epigenetics) AND (infection) AND (cytokine storm) |
|  | (Pérez-Novo and Bachert, 2015) | **25479316** | ("DNA methylation") AND ("airway") AND (inflammation) |
| **The epigenetic drift** | (Jones, Goodman and Kobor, 2015) | **25913071** | (epigenetics) AND ("DNA methylation") AND ("aging" OR "ageing") |
|  | (Zampieri *et al.*, 2015) | **25708826** | (epigenetics) AND ("DNA methylation") AND ("aging" OR "ageing") |
|  | (Issa, 2014) | **24382386** | ("aging" OR "ageing") AND ("epigenetic drift") |
|  | (Tan *et al.*, 2016) | **27498152** | ("aging" OR "ageing") AND ("epigenetic drift") |
|  | (Kochmanski *et al.*, 2017) | **27496716** | (DNA methylation) AND (epigenetic drift) AND (pollution) |
|  | (Capp and Thomas, 2021) | **33118188** | ("aging" OR "ageing") AND ("epigenetic drift") |
|  | (Zheng, Widschwendter and Teschendorff, 2016) | **27104983** | ("aging" OR "ageing") AND ("epigenetic drift") |
|  | (Vaiserman, 2018) | **30524474** | ("DNA methylation") AND ("aging" OR "ageing") AND ("epigenetic clock") |
|  | (Guillaumet-Adkins *et al.*, 2017) | **28808499** | (epigenetics) AND ("DNA methylation") AND ("aging" OR "ageing") |
|  | (Veitia *et al.*, 2017) | **27939088** | ("aging" OR "ageing") AND ("epigenetic drift") |
|  | (Tong *et al.*, 2024) | **38724732** | (epigenetics) AND ("DNA methylation") AND ("aging" OR "ageing") |
|  | (Keith Kelseyand Issa, 2022) | **Book chapter** | DNA methylation and aging |
|  | (Yan *et al.*, 2020) | **32991324** | (epigenetics) AND ("DNA methylation") AND ("aging" OR "ageing") |
|  | (Horvath, 2013) | 24138928 | (epigenetics) AND ("DNA methylation") AND ("aging" OR "ageing") |
|  | (Dutta *et al.*, 2024) | **38275598** | (epigenetics) AND ("DNA methylation") AND ("aging" OR "ageing") |
|  | (Slieker *et al.*, 2016) | **27654999** | (epigenetics) AND ("DNA methylation") AND ("aging" OR "ageing") |
|  | (Horvath and Raj, 2018) | **29643443** | ("DNA methylation") AND ("aging" OR "ageing") AND ("epigenetic clock") |
|  | (Mongelli *et al.*, 2023) | **36991505** | ("DNA methylation") AND ("aging" OR "ageing") AND ("epigenetic clock") |
| **Definition of the Epigenetic Biological Clock and implication in the Long COVID-19 syndrome Long COVID-19 syndrome** | (Perna *et al.*, 2016) | **27274774** | ("DNA methylation") AND ("aging" OR "ageing") AND ("epigenetic clock") |
|  | (Yusipov *et al.*, 2024) | 39002646 | ("DNA methylation") AND ("aging" OR "ageing") AND (epigenetic clock) |
|  | (Hao *et al.*, 2024) | 39251102 | (epigenetics) AND ("aging" OR "ageing") AND (epigenetic clock) |
| **Definition of the Epigenetic Biological Clock and implication in the Long COVID-19 syndrome Long COVID-19 syndrome** | (Crimmins *et al.*, 2021) | 33453106 | ("DNA methylation") AND ("aging" OR "ageing") AND (epigenetic clock) |
|  | (McCrory *et al.*, 2022) | 34999481 | ("DNA methylation") AND ("aging" OR "ageing") AND (epigenetic clock) |
|  | (Zhavoronkov and Mamoshina, 2019) | **31279569** | ("aging" OR "ageing") AND (longevity) AND (clock) AND (biomarkers) |
|  | (García-delaTorre *et al.*, 2024) | 38358578 | (epigenetics) AND ("aging" OR "ageing") AND (epigenetic clock) |
|  | (Declerck and Vanden Berghe, 2018) | **29337038** | ("DNA methylation") AND ("aging" OR "ageing") AND ("epigenetic clock") |
|  | (Nalbandian *et al.*, 2021) | 33753937 | (post-acute covid-19) AND (long covid) AND (clinical outcomes) AND (complications) AND (risks) |
|  | (Raciti *et al.*, 2014) | 24811791 | ("epigenetics") AND ("personalized medicine") AND ("diabetes") AND ("type 2") |
|  | (Stefansson and Esteller, 2013) | 23899662 | ("epigenetic modifications") AND ("cancer") AND ("personalized medicine") |
|  | (Brito and Noble, 2014) | 25249931 | (socioeconomic status) AND (brain development) |
|  | (Chen *et al.*, 2016) | 27690265 | ("DNA methylation") AND ("aging" OR "ageing") AND (epigenetic clock) |
|  | (Simons *et al.*, 2016) | 26765221 | (epigenetics) AND ("aging" OR "ageing") AND (epigenetic clock) |
|  | (McCrory *et al.*, 2019) | 30818253 | (epigenetics) AND (aging OR ageing) AND (epigenetic clock) AND (socioeconomic) |
|  | (Hughes *et al.*, 2018) | 30060108 | (DNA methylation) AND (age OR epigenetic clock) AND (socioeconomic) |
|  | (Justice and Kritchevsky, 2020) | 32515735 | (epigenetic biomarkers) AND (clinical trials) AND (aging OR ageing) |
|  | (Justice *et al.*, 2021) | 32902818 | (covid-19 OR sars-cov-2) AND (aging OR ageing) AND (geroscience) AND (disease) AND (infection) |
|  | (Martínez-Magaña *et al.*, 2024) | 38955096 | ("DNA methylation") AND ("aging" OR "ageing") AND (epigenetic clock) |
|  | (Kuo *et al.*, 2021) | 33684206 | (covid-19 OR sars-cov-2) AND ("aging" OR "ageing") AND (severity prediction) |
|  | (Maugeri *et al.*, 2024) | Book chapter | Epigenetics of aging and personalized intervention |
|  | (Wang et al., 2022) | 36336680 | (epigenetics) AND ("DNA methylation") AND ("aging" OR "ageing") |
|  | (Kikuchi *et al.*, 2022) | 36414620 | dnmt1[Title] |
| **Definition of the Epigenetic Biological Clock and implication in the Long COVID-19 syndrome Long COVID-19 syndrome** | (Galow and Peleg, 2022) | 35159278 | ("DNA methylation") AND ("aging" OR "ageing") AND (epigenetic clock) |
|  | (Corley *et al.*, 2021) | 33464637 | (acute covid-19 OR severe covid-19) AND (DNA methylation) |
|  | (Chamberlain *et al.*, 2023) | 37155825 | (covid-19 OR sars-cov-2) AND ("aging" OR "ageing") AND (severity prediction) |
|  | (Franzen *et al.*, 2021) | 34502212 | ("DNA methylation") AND ("aging" OR "ageing") AND (epigenetic clock) |
|  | (Bowler *et al.*, 2022) | 36261477 | (covid-19 OR sars-cov-2) AND (machine learning) AND (severity) |
|  | (Pang *et al.*, 2022) | 35719387 | ("DNA methylation") AND ("aging" OR "ageing") AND (epigenetic clock) |
|  | (Wang et al., 2022) | 35885892 | (covid-19 OR sars-cov-2) AND (epigenetics) |
|  | (Lee *et al.*, 2022) | 35798818 | (acute covid-19 OR severe covid-19) AND (DNA methylation) |
|  | (Xu *et al.*, 2022) | 36213637 | (epigenetics) AND ("aging" OR "ageing") AND (epigenetic clock) |
|  | (Calzari *et al.*, 2023) | **36800980** | (covid-19 OR sars-cov-2) AND (epigenetic drift) AND (clinic) |
|  | (Bejaoui *et al.*, 2023) | 38017502 | ("DNA methylation") AND ("aging" OR "ageing") AND (epigenetic clock) |
|  | (Balnis *et al.*, 2024) | 39024897 | (acute covid-19 OR severe covid-19) AND (DNA methylation) |
|  | (Calzari *et al.*, 2024) | 39164752 | ("DNA methylation") AND ("aging" OR "ageing") AND (epigenetic clock) |
| **COVID-19 and Epigenetic Drift** | (Loyfer *et al.*, 2023) | **36599988** | ("DNA methylation") AND ("cell types") AND ("human" OR homo sapiens) AND (atlas) |
|  | (Chlamydas, Papavassiliou and Piperi, 2020) | **32686577** | (covid-19 OR sars-cov-2) AND (epigenetic regulation) AND ("DNA methylation") |
| **The Clinical Epigenetics: Vision and Limits** | (Berdasco and Esteller, 2019) | **30479381** | (“clinical epigenetics”) AND (pathology OR disease OR cancer) AND (personalized medicine) AND (therapy OR treatment) |
|  | (Achinger-Kawecka *et al.*, 2024) | **38182927** | ("epigenetic therapy" OR "epigenetic therapies") |
|  | (Minetti *et al.*, 2006) | **16980968** | (dystrophy) AND (muscle) AND (therapy) AND (deacetylase inhibitors OR methyltransferase) |
|  | (Jones and Baylin, 2002) | **12042769** | (fundamental epigenetic events) AND (“cancer”) |
|  | (Marks and Breslow, 2007) | **17211407** | (histone deacetylase inhibitor) AND ("vorinostat") AND ("cancer") AND (drug) |
| **The Clinical Epigenetics: Vision and Limits** | (Rupaimoole and Slack, 2017) | **28209991** | (micro RNA "therapeutics") AND ("diseases") AND ("cancer") |
|  | (Horsthemke, 2022) | **35902960** | ("clinical epigenetics") AND ("epimutations") AND ("DNA methylation") |
|  | (Rauschert *et al.*, 2020) | **32245523** | (“clinical epigenetics”) AND (pathology OR disease OR cancer) AND (personalized medicine) AND (therapy OR treatment) |
|  | (Brookes and Shi, 2014) | **25195505** | ("epigenetic mechanisms") AND (chromatin) AND (disease OR "cancer") |
| **Reverting the clock** | (Fahy *et al.*, 2019) | **31496122** | (reverse) AND (epigenetic) AND (aging OR ageing) AND (regeneration) |
|  | (Galow and Peleg, 2022) | 35159278 | (reverse) AND (epigenetic) AND (aging OR ageing) AND (regeneration) |
|  | (Kriukov *et al.*, 2024) | **39072888** | (epigenetics) AND ("DNA methylation") AND ("aging" OR "ageing") AND (rejuvenation) |
|  | (Lu *et al.*, 2019) | **30669119** | (epigenetic) AND (GrimAge) AND (DNA methylation) AND (lifespan) |
|  | (Lu *et al.*, 2020) | **33268865** | (epigenetic) AND (aging OR ageing) AND (reverse) AND (reprogramming) |
| **Conclusions and perspectives** | (Attia, 2022) | 35598567 | (covid-19 OR sars-cov-2) AND ("aging" OR "ageing") AND (alterations) |
|  | (Humaira Amanullah *et al.*, 2024) | **38919619** | (covid-19 OR sars-cov-2) AND ("aging" OR "ageing") AND (alterations) |
|  | (Dawson and Kouzarides, 2012) | **22770212** | ("cancer epigenetics") AND (epigenetic mechanisms) AND ("therapy") |

**Table S2 Article research strategy.** The table indicates the relevant keywords used on the Pubmed database for searching the peer-reviewed articles published in English between 2020 and 2024, focusing on studies investigating the impact of SARS-CoV-2 infection on the human epigenome, listed according to the review’s sections.
